# First Molecular Survey of Equine Adenovirus Type 1 Infection Among Horses in Poland

**DOI:** 10.3390/ijms27156902

**Published:** 2026-08-01

**Authors:** Karol Stasiak, Magdalena Dunowska, Jerzy Rola

**Affiliations:** 1 Department of Virology and Viral Animal Diseases, National Veterinary Research Institute, Al. Partyzantow 57, 24-100 Pulawy, Poland; jrola@piwet.pulawy.pl; 2 School of Veterinary Science, Massey University, Palmerston North 4410, New Zealand; m.dunowska@massey.ac.nz

**Keywords:** EAdV-1, horses, quantitative PCR, sequence analysis, haplotype network

## Abstract

Equine adenovirus type 1 (EAdV-1) infection has been recognised among horses worldwide. The virus predominantly targets respiratory tract but has also been recovered from other body systems. The objective of the study was to determine the frequency of EAdV-1 infection among selected horse populations in Poland and to estimate genetic variability within the viruses obtained. A quantitative PCR assay targeting a partial *hexon* gene was used to screen 1150 nasal swabs collected from 1145 horses from 19 horse studs throughout Poland. The samples were collected opportunistically between 2012 and 2025. A set of PCR primers was designed to amplify and sequence a partial *fibre* gene from the qPCR-positive samples. Sequences from 1 to 5 clones from each PCR product were analysed using sequence identity matrix, phylogeny and median-joining haplotype networks. EAdV-1 was found in 49/1150 (4.3%) samples collected from 48 healthy horses and one horse showing clinical signs of respiratory disease. Partial *fibre* gene sequences from individual horses were closely related to each other. Sequences from each stud were closely related, even if they were collected over several years. Taken together, this study provides first insights into the epidemiology of active infections caused by EAdV-1 among horses from selected Polish studs, including genetic variability among the viruses detected.

## 1. Introduction

Adenoviruses comprise a group of pathogens that infect animals from many species. To date, they have been identified in a variety of hosts including birds (ducks, fowls, geese, pigeons, turkeys), amphibians (frogs), fish (white sturgeons), reptiles (lizards, snakes) and mammals (bovines, sheep, deer) including horses [1]. Among all members of the *Adenoviridae* family and *Mastadenovirus* genus, equine adenovirus type 1 (EAdV-1) and type 2 (EAdV-2) are known to infect horses [2]. While EAdV-1 has predominantly been associated with the upper respiratory tract infection, EAdV-2 usually infects the gastrointestinal tract [3]. These viruses represent two different species: EAdV-1 (species *Mastadenovirus equi*) and EAdV-2 (species *Mastadenovirus equidae*) [4].

EAdV-1 has been isolated from nasal swabs collected from foals, young and adult horses with and without clinical signs of respiratory disease [5,6,7]. The clinical signs, if present, include nasal discharge, coughing after exercise, conjunctivitis, submandibular lymphadenopathy and pyrexia [3]. The most serious disease, albeit rare, linked to adenoviral infection is severe bronchopneumonia in Arabian and Thoroughbred foals [6,8].

Evidence based on the results of a panel of different serological assays indicates that EAdV-1 has a worldwide distribution, with reported seroprevalence of 39.0% in the Netherlands [9], 10.6% in Ireland [10], 24.1% in the United Kingdom [11], 54.9% to 77% in Australia [12,13], 39.0% to 91.0% in New Zealand [14,15], 8.6% to 82.7% in Japan [16,17], and 4.5% in Nigeria [18].

Thus, based on serological evidence, EAdV-1 infections appear to be common among horse populations around the world. However, there are relatively few studies on the detection of active adenoviral infections and hence, relatively little is known about the epidemiology of these viruses. To date, such studies have been conducted in Belgium [19], France [20], Turkey [21], the Republic of Korea [22], Australia [23] and the United States [5], with reported frequences of infection ranging from 0% to 4.0% among adult horses. The sampling strategy and populations sampled varied between studies, but EAdV-1 was detected in samples from both healthy and diseased horses in those studies which included both categories [5,20,22]. Only two papers included phylogenetic analyses of a small number (*n* = 8) of EAdV-1 sequences, with overall similarity ranging between 98.8% to 100% based on a 265 bp fragment of the *hexon* gene [21,22].

Previously, we have performed two surveys on equid herpesviruses and equine rhinitis viruses among Polish horses of various breeds and ages [24,25]. The purpose of the current study was to investigate the prevalence of EAdV-1 infections among horses at selected Polish national studs and to estimate their genetic variability, hence expanding the previously published data to EAdV-1 infections.

## 2. Results

### 2.1. Infection with EAdV-1

**Figure 1:**
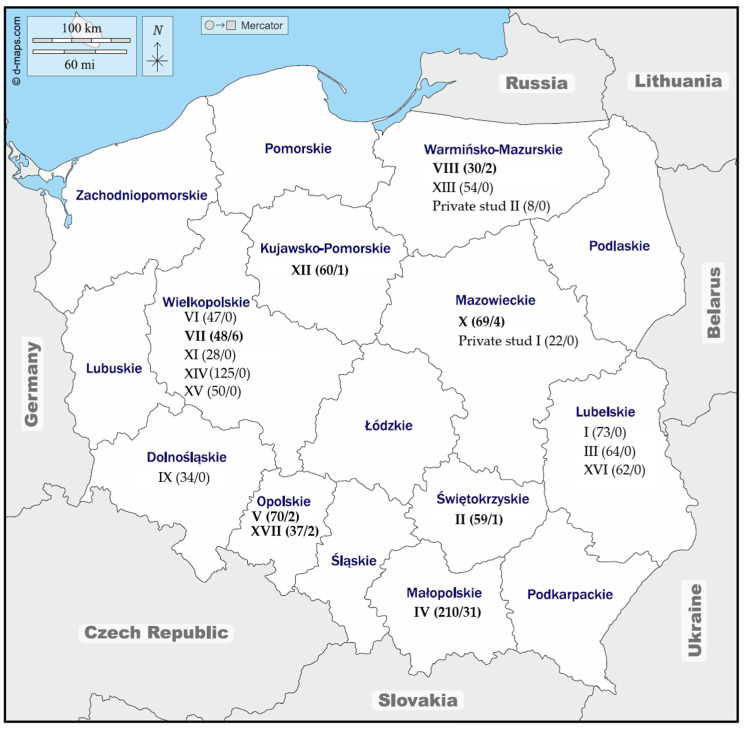
Map of Poland showing the location of the horse studs (denoted by Arabic numbers). The number of samples collected from each stud/the number of EAdV-1-positive samples are shown in the brackets. Available from: https://www.d-maps.com/carte.php?num_car=4308&lang=en, accessed on 28 February 2026.

**Figure 2:**
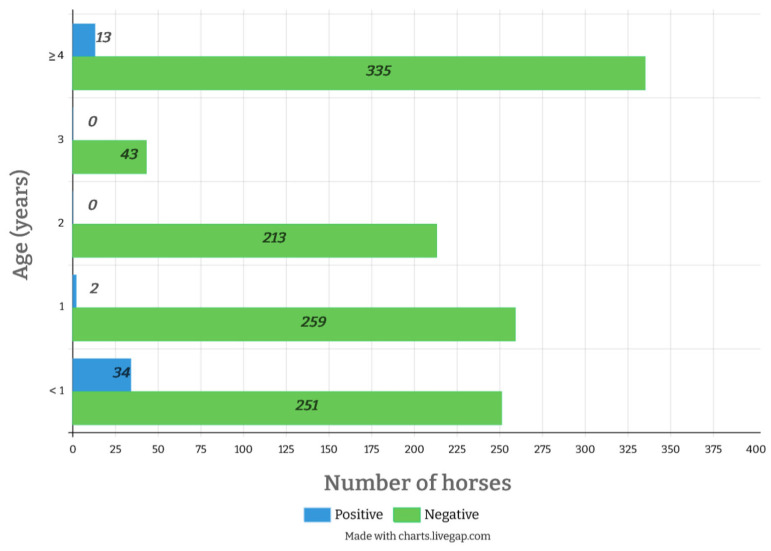
Frequency of detection of equine adenovirus type 1 in nasal swabs from all horse-sampling occasions (*n* = 1150), stratified by age group. The “positive” and “negative” descriptors relate to results of EAdV-1-specific quantitative PCR.

Equine adenovirus 1 DNA was detected in 49/1150 (4.3%) samples tested. The positive horses were from 8/19 studs: II (*n* = 1), IV (*n* = 31), V (*n* = 2), VII (*n* = 6), VIII (*n* = 2), X (*n* = 4), XII (*n* = 1) and XVII (*n* = 2) (Figure 1). Out of the 99 horses sampled with clinical signs of respiratory disease, EAdV-1 DNA was found in only one (1%). The remaining 48 EAdV-1 positive swabs (*n* = 1051) came from healthy horses, which constituted 4.6% of those tested. The highest number of EAdV-1-positive horses were Hucul horses (stud IV), but the proportion of virus-positive horses varied between individual samplings at that stud. Out of all the age groups tested, foals were most likely to be positive for EAdV-1 (11.9%; 95% CI: 8.7–16.2), followed by the group of the oldest horses (3.7%; 95% CI: 2.2–6.3), and yearlings (0.8%; 95% CI: 0.1–2.8) (Table 1). None of the two- or three-year-old horses were shedding EAdV-1 at the time of sampling (Figure 2). The median age of EAdV-1-positive horses in the oldest group was 10 years (range 4–13 years), while the median age of all the horses sampled in that age category was nine (range 4–28).

### 2.2. Molecular and Phylogenetic Analysis

Long PCR products were amplified from selected 19 EAdV-1 qPCR-positive samples. The selected samples included all samples with a Cq value of ≤33 from all studs but stud IV. From that stud, nine EAdV-1 positive samples were chosen by convenience from 20 with Cq ≤ 33. Sequence alignments of the partial *fibre* gene from all clones showed 97.7% to 100% identity at the nucleotide level and 96.4% to 100% identity at the amino acid level. The degree of identity between Polish EAdV-1 sequences and the two international sequences available in the GenBank ranged between 97.6% and 99.5% at the nucleotide level and 97.1% to 100% at the amino acid level. The highest genetic variability was observed between sequences from stud IV (97.8% to 100% at the nucleotide level), represented by 36 clones obtained from nine Hucul horses (Table 2 and Figure S1).

**Figure 3:**
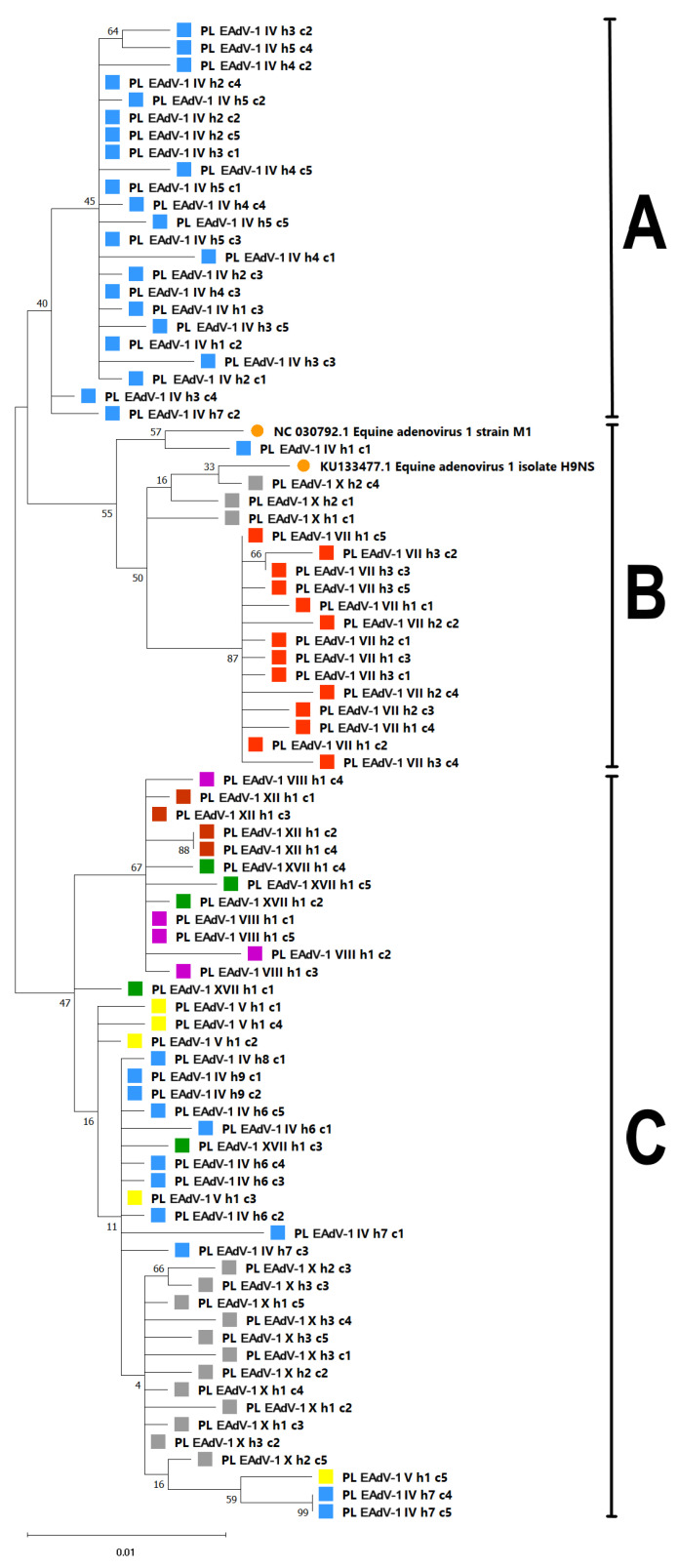
Phylogenetic tree of equine adenovirus type 1 (EAdV-1) based on 841 bp fragment from *fibre* protein (nt 718–1559 in EAdV-1 accession number NC_030792.1). The sequences comprise Polish EAdV-1 sequences of clones (*n* = 84) from 19 PCR products obtained from 19 horses from seven studs. Sequences from the same stud are indicated by squares of the same colour. International sequences (*n* = 2) were obtained from GenBank (orange circles). The Polish sequences analysed in the current study are labelled PL_EAdV-1_stud number_horse number_clone number. The clusters (**A**–**C**) are shown on the right. The evolutionary history was inferred using the maximum likelihood method with a bootstrap value of 1000 and the Tamura model. The tree with the highest log likelihood (−2354.08) is shown. The evolutionary rate differences among sites were modelled using a discrete gamma distribution across five categories (+G, parameter = 0.5560). The complete deletion option was applied to eliminate positions containing gaps and missing data. Evolutionary analyses were conducted in MEGA12 [26].

Based on the phylogenetic tree, Polish EAdV-1 sequences clustered within three main clusters, although with a relatively weak bootstrap support (40% to 55%) for such groupings (Figure 3). While sequences from the same horse were often identical or clustered together, that was not always the case. For example, the sequence obtained from clone 1 from horse 1 (PL_EAdV1_IV_h1_c1) at stud IV appeared to be more closely related to the Australian reference strain M1 than the remaining sequences from the same horse and other horses from this stud. Similarly, EAdV-1 sequences from horse 2 at stud X were distributed throughout the tree in clusters B and C.

### 2.3. Haplotype Network Analysis

**Figure 4:**
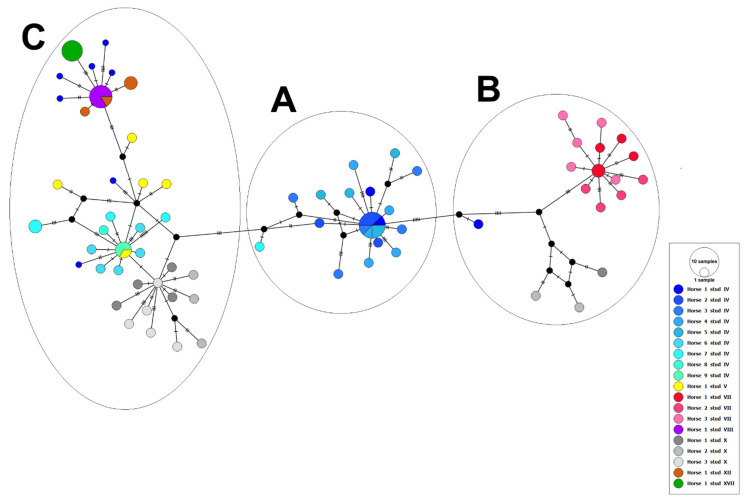
Haplotype network analysis based on 84 sequences of the partial *fibre* protein gene of equine adenovirus type 1 (EAdV-1) obtained from horses at Polish studs. The median-joining haplotype network comprised 70 haplotypes. It was drawn using PopART version 1.7 (available from http://popart.maths.otago.ac.nz) with default parameters. Nodes are scaled based on the number of sequences and coloured by individual horses from which they were obtained. Horses from the same stud are shown in different shades of the same colour. Small closed black circles indicate inferred nodes that are not represented among sequences included in the network. The AMOVA results corresponding to this network are shown in Table 3. The clusters (**A**–**C**) are shown at the top of the network.

**Figure 5:**
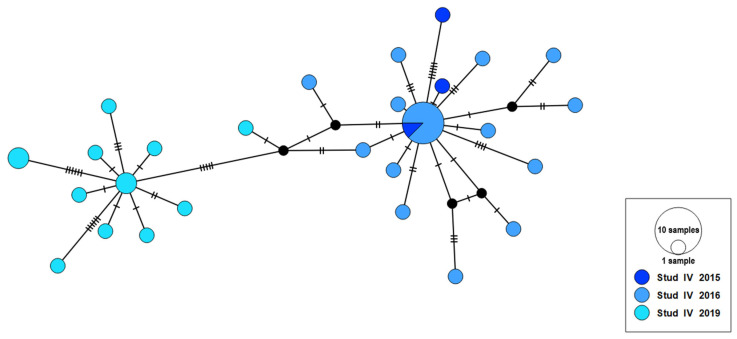
Median-joining haplotype network based on the partial *fibre* protein gene of EAdV-1 sequences from stud IV. Nodes are scaled based on the number of sequences and coloured by year of isolation. Small closed black circles indicate inferred nodes that are not represented among sequences included in the network.

The 84 Polish EAdV-1 partial *fibre* protein sequences (841 bp) formed 70 haplotypes. The median-joining haplotype network of the genetic structure amongst sequences showed a strong correlation between EAdV-1 sequences and individual horses (PhiST = 0.76, *p* < 0.001) (Figure 4). In addition, there was some structure visible in the network when sequences were coloured by the stud from which the samples were obtained (PhiST = 0.59, *p* < 0.001) (Table 3). The clustering of EAdV-1 haplotypes did not correlate with the year of virus detection (PhiST = 0.08, *p* = 0.001) when all sequences were considered. However, when the analysis was restricted to stud IV, as the only stud from which EAdV-1 sequences were available from three different years, there was some temporal structure in the network, with 63.9% of the variability attributed to differences between populations (PhiST = 0.63, *p* < 0.001). This indicates that viral sequences obtained within the same year were more similar to each other than to the sequences obtained in different years on that stud (Figure 5).

## 3. Discussion

The aim of the study was to determine the frequency of EAdV-1 shedding and to analyse the viral sequences from diverse populations of horses in Poland. Only 4.3% of 1150 nasal swab samples collected from 1145 horses included in the study were positive for EAdV-1. We do not have the corresponding serology data to determine the true extent of EAdV-1 infection among the horses sampled, but older serological studies suggested a high frequency of EAdV-1 infection within the first year of life, often in the presence of maternal antibodies, with overall seroprevalence of around 70% [15,27]. Despite the high frequency of infection suggested by serological data, the rates of adenovirus detection, either by culture or by molecular methods, were often low, similar to the results of the current study. No adenoviruses were cultured from nasal swabs of foals and horses from outbreaks of respiratory disease in New Zealand [15,28], and nasal swabs from only 1.4% of 359 horses in Korea tested positive for EAdV-1 DNA [22]. A comparatively higher (16/52, 30%) frequency of EAdV-1 detection was reported among a group of 52 racehorses in training in France [20]. However, these horses were sampled monthly for two years, and the overall prevalence of EAdV-1 detection among all samples collected was 3.6%, comparable to the current results. Thus, it appears that EAdV-1 can be maintained within a population with only a small proportion of horses actively infected with the virus at any given time.

The frequency of EAdV-1 detection at stud IV varied considerably between years. The 2018 testing results were similar to those obtained in 2015 and 2017, with only a few (or none) horses testing positive for EAdV-1. The 2016 and 2018 results stood up, as a considerably higher proportion of horses tested were positive for the virus. It is difficult to offer a plausible explanation for these differences without a more detailed epidemiological investigation. It may be EAdV-1 infection is somewhat cyclical, with more horses infected when the herd immunity diminishes. However, there is little to no data available for the length of protective immunity to EAdV-1 following infection. In one longitudinal study [29], reinfection was common, often in the presence of EAdV-1 antibodies, which does not seem to support this theory. These results could also simply reflect the selection of horses. The Hucul horses are maintained and housed in breeding groups, so it is possible that there are differences in the circulation of viruses between different groups. Finally, since we used archival samples, we cannot fully exclude the possibility that 2018 lot of samples was mistreated in some way during transport or storage, which affected the quality of the samples.

Out of four age groups included in the current study, foals were the most likely to be shedding EAdV-1. The high frequency of EAdV-1 infection among foals has been suggested by others. In one study, EAdV-1 was isolated from a healthy 3-day-old foal [27]. In another study, EAdV-1 was detected from nasal swabs of 40% hospitalised neonatal foals and 58% of healthy neonatal foals within the first 24 h of life [5]. Although that study involved a small number of foals that were all born in one US institution, the timing of development of adenovirus-associated severe fatal bronchopneumonia in Arabian foals affected by primary severe combined immunodeficiency (PSCID) syndrome also supports frequent infections with EAdV-1 within the first month of life. These foals typically succumb to disease soon after they lose maternal antibodies, at around 2 to 5 months of age [30].

This is also consistent with results of studies that used serology for detection of EAdV-1 infection. In one such study, EAdV-1 antibodies were detected in 21/23 (91%) foals in New Zealand on at least one monthly sampling occasion in their first 13 months of life, and 16/23 (69%) foals showed serological evidence of recent EAdV-1 infection during that time. In another multi-country study, the prevalence of EAdV-1 antibodies was highest in foals younger than 5 months of age (22/25, 88%), then declined slightly between five and 12 months of age (90/147, 61%), and increased again (349/459, 76%) for horses older than one year of age, suggesting that many EAdV-1 infections occurred within the first year of life [27].

All but one of the horses that were EAdV-1-positive were healthy at the time of sampling, suggesting that EAdV-1 was not an important contributor to the respiratory disease observed among sampled horses at selected studs (Table 1). This is similar to the findings from the Korean study, where only 1/5 EAdV-1-positive horses was affected by overt respiratory disease [22]. Others also concluded that EAdV-1 was rarely associated with respiratory disease, as nasal swabs from only 4/103 (4%) horses with respiratory disease in Belgium [19] and 1/73 horses with respiratory disease in Turkey [21] were positive for EAdV-1 DNA at the time of sampling. Similar conclusions were reached based on the lack of serological evidence of recent EAdV-1 infection among horses with respiratory disease in New Zealand [28]. However, others reported isolation of EAdV-1 from cases of respiratory disease and experimental infection with EAdV-1 produced respiratory disease of various severity [7,8,31]. The virus also causes severe fatal pneumonia in immunocompromised foals, including Arabian foals with PSCID as well as possibly foals from Fell and other native pony breeds in the United Kingdom affected by an inherited immunodeficiency syndrome [30,32,33]. By extrapolation, it may be that EAdV-1 infection causes overt disease only in horses that are transiently immunosuppressed by factors such as stress or concurrent infections. Finally, we cannot exclude the possibility that horses infected with EAdV-1 in the current study experienced some respiratory impairment, but it was subtle enough not to be apparent without more targeted investigation including modalities such as endoscopy or exercise challenge.

The results may also reflect the timing of sample collection. Because nasal swabs from most diseased horses were collected by attending veterinarians, some horses may have no longer been in the early stages of infection, when detection of respiratory viruses is most likely. The duration of EAdV-1 nasal shedding has not been well characterised. After experimental infection with EAdV-1, the virus was isolated from nasal swabs for six to 18 days in one study [34,35], although it was detected from nasopharyngeal mucus for up to 68 days in another [29]. Despite the differences in the reported duration of shedding, it seems likely that EAdV-1 would have been detected in more than one nasal swab collected from diseased horses had the virus been aetiologically involved in the respiratory disease experienced by the horses sampled. This is further strengthened by the fact that EAdV-1 sequences were detected in samples from 48 healthy horses even though these were first screened and pooled, unlike samples from diseased horses, which were all tested individually. This testing strategy may have led to under-detection of low-level shedders in the healthy horse group compared with the diseased group.

The partial sequence of the *fibre* gene was used to estimate the genetic variability within Polish EAdV-1 sequences. The primary role of the *fibre* protein is to tether the viral capsid to the cell surface and to initiate entry to the host cell via interactions with a cellular receptor [36]. As such, several authors described regions of high variability within this gene [37,38]. At the time of commencement of the current study, only two full genomic sequences of EAdV-1 were available in GenBank. Based on the comparison of these sequences, the *fibre* gene was chosen for phylogenetic analysis, as it contained the highest number of single nucleotide changes within a defined region. However, recently published data suggest that the *fibre* gene of EAdV-1 is more conserved than the *hexon* gene [39], and hence, the *fibre* gene may not have been the best choice for phylogenetic analysis. *Hexon* and *fibre* gene sequences of only six viruses were compared in that study, so our data add further information on the stability of the *fibre* gene using a considerably higher number of viral sequences.

A relatively strong correlation between genetic structure and the stud from which nasal swabs were collected suggests that there were barriers to EAdV-1 spread between studs and that infections were maintained among horses at the studs. This is consistent with the distance between these studs of several hundred kilometres (different provinces), with the closest studs (V and XVII from Opolskie province) separated by more than 20 km. Factors that likely facilitated circulation of EAdV-1 at each stud include relatively high environmental stability of adenoviruses [40,41] and their tendency to establish persistent infections in lymphoid tissues [42], although the latter has not been shown specifically for EAdV-1.

Nonetheless, some genotypes were shared between horses from different studs. For example, a cluster in the top left corner of the network (Figure 4) comprised EAdV-1 sequences from four horses from four different studs (IV, VIII, XII and XVII). As these studs were located in different regions of Poland and bred different types of horses, it is unlikely that this represented epidemiological links. It is more likely that this particular genotype may be common among various populations of horses in Poland. The clustering within the network could not be explained by the year of sampling (Table 3), although there was some indication of temporal clustering of viral sequences from stud IV (Figure 5). Overall, our results suggest persistence and high genetic stability of EAdV-1 at the studs sampled. While limited comparable data are available for equine adenoviruses, this is consistent with high genetic stability of human adenoviruses (HAdV) reported by several authors. In one study, two most common lineages of HAdV-A31 were shown to have co-circulated in Germany for at least 59 years [43], and selected HAdV-55 genotypes were shown to persist in some geographical locations for years [44]. EAdV-1 was detected in samples collected from more than one horse at three studs (IV, VII, and X). While all the sequences from stud VII clustered together, sequences from studs X and IV were present in two or three clusters located in different parts of the network, respectively. The highest genetic variability was observed among EAdV-1 sequences from stud IV, which may simply reflect the highest frequency of EAdV-1 detection at that stud, with a proportion of sampled horses testing positive for EAdV-1 on 4/5 sampling visits (Table 1). The reason for such a relatively high frequency of EAdV-1 detection among horses from stud IV remains unclear. That stud breeds horses of a native Hucul breed, with most of the horses managed in groups, under conditions close to their natural environment. This is one factor that may have facilitated spread of EAdV-1 among horses at stud IV. In addition, it is also possible that some breed-specific predispositions exist, although none of the EAdV-1-positive horses from stud IV showed any signs of overt clinical disease at the time of sampling. We have presented the largest and most comprehensive study of genetic variability between EAdV-1 sequences. Our dataset included 19 viruses obtained from horses of different ages and breeds from various regions in Poland. To our knowledge, this is the first study reporting the presence of multiple EAdV-1 sequence variants among cloned amplicons derived from individual clinical samples. The virus sequences obtained from the same horse were homogenous and more similar to each other than to sequences from a different horse. However, the limited number of clones analysed did not permit a comprehensive assessment of EAdV-1 quasispecies. The study had several other limitations. As most of the horses were sampled only once, the persistence of EAdV-1 infection within individual horses could not be assessed. The sampling strategy was opportunistic and included both routine surveillance samples and samples collected during diagnostic investigations of respiratory disease. Consequently, the study population cannot be considered representative of the entire Polish equine population. Finally, sequence analyses were limited to a partial *fibre* gene. Sequencing of the whole viral genomes would have been preferred but was beyond the financial scope of the study.

## 4. Materials and Methods

### 4.1. Sample Collection

A total of 1150 nasal swab samples collected from 1145 horses from 17 national Polish horse studs and two small private studs were analysed in the study (Table 1). The samples comprised archival samples that were collected between November 2012 and April 2025 as part of the national surveillance programme, in addition to samples submitted by local veterinarians as part of diagnostic investigation of the causes of respiratory disease at selected studs. Some of the samples had been previously tested for equine herpesviruses [24] and equine rhinitis viruses [25]. Whenever feasible, horses of different ages were sampled at each stud visit (Figure 1). With the exception of four mares from stud IV (M1, M2, M6, and M7) which were sampled on more than one occasion (M1, M2 and M6—twice, M7—three times) during four visits to that stud, all other horses were sampled once. As the interval between sampling visits at stud IV was approximately one year, the samples collected from these mares were treated as separate epidemiological observations for analyses of EAdV-1 detection. The health status of horses was noted at the time of sampling. Horses were considered to be affected by respiratory disease if they showed overt clinical signs such as coughing or nasal discharge. Rectal temperature was not taken. All samples were delivered to the Department of Virology and Viral Animal Diseases in Pulawy (Poland) on ice packs within 24–48 h from collection.

### 4.2. Processing of Samples

Upon arrival, each swab was vortexed for 30 s to release viruses into the transport medium and then discarded. Pooled samples were created using a portion (20–30 µL) of each transport medium from seven to 12 healthy animals from the same stud and age group. Swabs from horses with clinical signs of respiratory disease were processed individually without pooling. All samples were stored at −80 °C for further processing. Viral DNA was extracted from both individual and pooled samples using the QIAamp DNA Mini Kit (Qiagen, Hilden, Germany), according to the manufacturer’s instructions, and eluted with 50 µL of the supplied elution buffer. All extraction runs included positive (field isolate of EAdV-1) and negative (EAdV-1 negative transport medium) extraction controls.

### 4.3. Quantitative PCR

Amplification of viral DNA was performed by quantitative PCR (qPCR) assay targeting the highly conserved fragment of the *hexon* gene of EAdV-1, as described by Doubli-Bounoua [20]. The limit of detection of the assay in our hands was 10 copies of EAdV-1 target per µL of template. Each virus-specific reaction consisted of 2 µL of extracted DNA, forward and reverse primers (Table 4) at a final concentration of 400 nM each and 200 nM of the probe in 1x TaqMan^®^ Universal PCR Master Mix (Thermo Fisher Scientific, Warrington, UK). The following cycle conditions were used: uracil-DNA glycosylase treatment at 50 °C for 2 min, initial denaturation at 95 °C for 10 min, followed by 40 cycles of 95 °C for 15 s and 55 °C for 1 min. Positive (EAdV-1 DNA extracted from a field virus) and negative (water) controls were included in each run. All reactions were performed using a LightCycler^®^96 System (Roche, Mannheim, Germany). Samples that produced Cq values below 37.0 were considered positive for EAdV-1 DNA. All swabs from negative pools were considered negative for EAdV-1. Samples from pools that returned positive qPCR results were retested individually.

### 4.4. Conventional PCR and Cloning

Viral DNA from selected (*n* = 19) EAdV-1 positive swabs was used as a template in a conventional PCR assay targeting a fragment of the *fibre* gene of EAdV-1, with the expected product size of 861 bp using newly designed primers (Table 4). Each PCR reaction contained 600 nM of each primer, 1 x buffer (Sigma-Aldrich, St. Louis, USA), 0.2 mM deoxynucleotide mix (Sigma-Aldrich), 0.5 µL JumpStart AccuTaq LA DNA Polymerase (Sigma-Aldrich), and 2 µL of template DNA in a total volume of 25 µL. Amplifications were performed in a Biometra Thermocycler (Biometra, Göttingen, Germany) using the following cycling conditions: initial denaturation 5 min at 94 °C, followed by 40 cycles of denaturation (1 min at 94 °C), primer annealing (1 min at 59.0 °C), elongation (1 min at 72 °C) and the final elongation step (5 min at 72 °C). Amplified products were subjected to electrophoresis through a 1.5% GelRed stained agarose gel, purified with GeneJET PCR Purification Kit (Thermo Scientific, Carlsbad, CA, USA) and ligated into TOPO TA Cloning Kit for Sequencing (Thermo Fisher Scientific, Carlsbad, CA, USA) according to the manufacturer’s instructions. Ligated products were chemically transformed into *E. coli* JM109 Competent Cells (Promega Corporation, Madison, WI, USA). Plasmid DNA was isolated from up to five insert-positive clones using PureLink™ Quick Plasmid Miniprep Kit (Thermo Fisher Scientific, Vilnius, Lithuania) according to the manufacturer’s instructions and sequenced with the same primers used for amplification using BigDye^®^ Terminator version 3.1 (Applied Biosystems, Vilnius, Lithuania) on a 3730xl DNA Analyzer at the Genomed (Warsaw, Poland).

### 4.5. Sequence Analyses

The forward and reverse sequences obtained from each recombinant *E. coli* colony were trimmed to an identical length (841 bp) and assembled using BioEdit software v.7.2.5. The consensus sequences were aligned with the reference sequence of EAdV-1 strain M1 (GenBank accession number NC_030792.1) using the MUSCLE algorithm from the Molecular Evolutionary Genetics Analysis software package, version 12 (MEGA12) [26]. Identity among sequences was calculated using the “Sequence Identity Matrix” formula in BioEdit software v.7.2.5 and illustrated as heatmap using a Sequence Demarcation Tool Version 1.3 (SDTv1.3) [45]. Phylogenetic tree was constructed using the maximum likelihood method with 1000 bootstrap replicates using the T92+G substitution model in MEGA12. All nucleotide sequences were used as an input file to generate a haplotype list in the DnaSP software (version 6.12.03, available from https://www.ub.edu/dnasp/ (accessed on 4 February 2026)). The haplotypes were then exported in the nexus format with trait blocks added to represent breed, stud number, each individual horse and age group of the sampled animals. The median-joining haplotype networks were drawn using default parameters in PopART version 1.7 (available from http://popart.maths.otago.ac.nz). Analysis of molecular variance (AMOVA within PopART) was used to test for correlation between the genetic structure of the EAdV-1 sequences and selected traits. The strength of correlation was represented by a PhiST value, with 0 indicating no correlation and 1 indicating perfect correlation. The corresponding *p*-values were generated by reference to 1000 random permutations of the input data.

### 4.6. GenBank Accession Numbers

The nucleotide sequences of Polish EAdV-1 described in this study were submitted to GenBank under the following accession numbers: PZ398286–PZ398369.

## 5. Conclusions

We have reported for the first time the detection of EAdV-1 in Poland. In addition, we have generated and analysed the largest dataset of EAdV-1 sequences. The results of the present study demonstrate that the proportion of horses shedding EAdV-1 was low within the populations sampled and that shedding of the virus was not associated with overt respiratory disease. The frequency of EAdV-1 detection was highest in foals, in agreement with previously published data. The viral sequences appeared stable within individual horses. Some genotypes were stud-specific, others were detected at more than one stud. The *fibre* gene sequence showed little variability between different genotypes and hence, it may be worthwhile investigating the variability of other genomic regions in future studies.

## Figures and Tables

**Table 1:** Incidence of equine adenovirus 1 (EAdV-1) infection among horses sampled at each sampling visit between November 2012 and April 2025 at each of the 19 Polish studs included in the study. Confidence intervals (CI) were calculated using Wilson/Brown method. #–number.

Stud Farm	Breed	Horses [*n*]	Disease [*n*]	Region	Sampling Date	Positives #	Positives % (95% CI)
I	Arabian	19	19	Lubelskie	Apr 2015	0	0% (0–16.8)
		27	27		Sep 2018	0	0% (0–12.5)
		27	0		Oct 2024	0	0% (0–12.5)
II	Arabian	19	19	Swietokrzyskie	Apr 2015	1	5.3% (0.3–24.6)
		40	0		Apr 2024	0	0% (0–8.8)
III	Arabian	15	15	Lubelskie	Feb 2016	0	0% (0–20.4)
		49	0		May 2024	0	0% (0–11.7)
IV	Hucul horses	45	0	Małopolskie	May 2015	4	8.9% (3.5–20.7)
		39	0		May 2016	12	30.8% (18.6–46.4)
		50	0		May 2017	2	4% (0.7–13.5)
		44	0		May 2018	0	0% (0–8)
		32	0		May 2019	13	40.6% (25.5–57.7)
V	Malopolska horse	40	0	Opolskie	Apr 2016	2	5% (0.9–16.5)
		30	0		Mar 2025	0	0% (0–11.4)
VI	Wielkopolska horse	47	0	Wielkopolskie	May 2016	0	0% (0–7.6)
VII	Wielkopolska horse	48	0	Wielkopolskie	May 2016	6	12.5% (5.9–24.7)
VIII	Wielkopolska horse	30	0	Warminsko-mazurskie	Apr 2016	2	6.7% (1.2–21.3)
IX	Silesian horse	34	0	Dolnoslaskie	Apr 2016	0	0% (0–10.2)
X	Thoroughbred	60	1	Mazowieckie	Apr 2016	4	6.7% (2.6–15.9)
		9	0		Apr 2025	0	0% (0–29.9)
XI	Thoroughbred	10	10	Wielkopolskie	Apr 2014	0	0% (0–27.8)
		18	0		Apr 2025	0	0% (0–17.6)
XII	Polish Coldblood	60	0	Kujawsko-pomorskie	Apr 2016	1	1.7% (0.1–8.9)
XIII	Polish Konik	54	0	Warminsko-mazurskie	Jun 2015	0	0% (0–6.6)
XIV	Polish Konik	61	0	Wielkopolskie	May 2016	0	0% (0–5.9)
		64	0		May 2017	0	0% (0–5.7)
XV	Polish Konik	50	0	Wielkopolskie	May 2016	0	0% (0–7.1)
XVI	Polish Konik	62	0	Lubelskie	May 2016	0	0% (0–5.8)
XVII	Polish Halfbred horse	37	0	Opolskie	Apr 2016	2	5.4% (1–17.7)
Private stud I	Thoroughbred	22	0	Mazowieckie	Nov 2012	0	0% (0–14.9)
Private stud II	Polish Konik	8	8	Warminsko-mazurskie	Jun 2015	0	0% (0–14.5)
**Total**		**1150**	**99**			**49**	**4.3% (3.2–5.6)**

**Table 2:** Percentage nucleotide identity among clones derived from the same amplicon of a partial *fibre* gene sequence of equine adenovirus 1, and the range of identities between clones from different amplicons. Each amplicon represents EAdV-1 detected in the nasal swab of an individual horse.

Sequence ID	Stud Farm	No. of Clones Sequenced	% Nucleotide Identity	
			Within Clones	Among Horses
PL_EAdV1_IV_h1	IV	3	99.1–99.8	97.8–100
PL_EAdV1_IV_h2		5	99.8–100	
PL_EAdV1_IV_h3		5	99.1–99.7	
PL_EAdV1_IV_h4		5	99.4–100	
PL_EAdV1_IV_h5		5	99.4–100	
PL_EAdV1_IV_h6		5	99.5–99.7	
PL_EAdV1_IV_h7		5	98.5–100	
PL_EAdV1_IV_h8		1	-	
PL_EAdV1_IV_h9		2	100	
PL_EAdV1_V_h1	V	5	99.1–99.7	-
PL_EAdV1_VII_h1	VII	5	99.5–100	99.4–100
PL_EAdV1_VII_h2		4	99.2–99.6	
PL_EAdV1_VII_h3		5	99.2–99.7	
PL_EAdV1_VIII_h1	VIII	5	99.2–100	-
PL_EAdV1_X_h1	X	5	98.2–99.7	98.2–99.6
PL_EAdV1_X_h2		5	98.4–99.5	
PL_EAdV1_X_h3		5	99.2–99.7	
PL_EAdV1_XII_h1	XII	4	99.6–100	-
PL_EAdV1_XVII_h1	XVII	5	98.8–99.6	-

**Table 3:** Analysis of molecular variance (AMOVA) results indicating the strength of correlation (PhiST) between population genetic structure within Polish equine adenoviruses and selected traits based on partial sequence of *fibre* protein.

Test	Variation Within Populations	Variation Between Populations	PhiST	*p*-Value
Stud ^1^	40.2%	59.8%	0.59	<0.001
Individual horse	23.9%	76.1%	0.76	<0.001
Year of isolation ^2^	92.2%	7.8%	0.08	0.001
Year of isolation (Stud IV)	36.1%	63.9%	0.63	<0.001

^1^ IV; V; VII; VIII; X; XII; XVII; ^2^ 2015; 2016; 2019.

**Table 4:** Primers and probes used for detection of equine adenovirus type 1 among Polish horses.

Assay	Region	Primers/Probes (5′ to 3′)	Size (bp)	Reference
Quantitative PCR	Hexon	Forward: GATGCTTCCACAATGGTCCT	163	[20]
		Reverse: CTCGGTGGTGACATCGTG		
		Probe: FAM-GAATACCTGTCGCCCGCTC-BHQ1		
Conventional PCR	Fibre	Forward: GGGGACGTGCTCAAGGTAAA	861	[this study]
		Reverse: GGCAATGGTGATGCGATTGT		

## Data Availability

The nucleotide sequences described in this study were submitted to GenBank under the following accession numbers: PZ398286–PZ398369.

## Supplementary Materials

The following supporting information can be downloaded at https://www.mdpi.com/article/10.3390/ijms27156902/s1.
