# Supplementary material for: First Molecular Survey of Equine Adenovirus Type 1 Infection Among Horses in Poland
Source: Int J Mol Sci. 2026 Aug 1;27(15):6902. doi: 10.3390/ijms27156902 (PMC13467118; doi:10.3390/ijms27156902)
Supplement: Supplementary file 1 [file ijms-27-06902-s001.zip › ijms-4406601-supplementary.pdf]

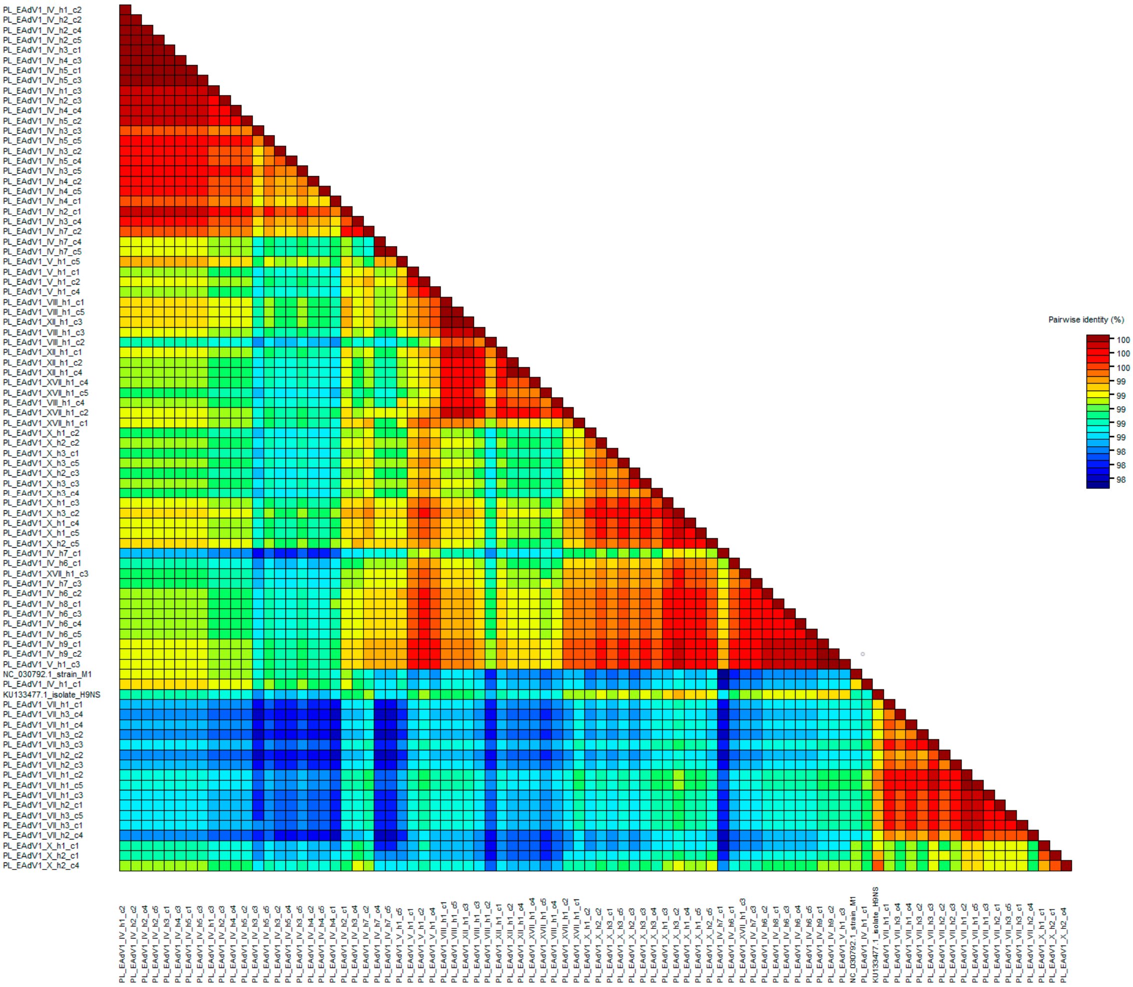

Figure S1. Identity matrix for equine adenovirus type 1 (EAdV-1) used in the study. Heatmap showing percent of identity between pairs of EAdV-1 partial *fibre* gene.
